# Flexible Biosensors Based on Colorimetry, Fluorescence, and Electrochemistry for Point-of-Care Testing

**DOI:** 10.3389/fbioe.2021.753692

**Published:** 2021-09-28

**Authors:** Tingyi Yan, Guangyao Zhang, Huining Chai, Lijun Qu, Xueji Zhang

**Affiliations:** ^1^ State Key Laboratory of Bio-Fibers and Eco-Textiles, Intelligent Wearable Engineering Research Center of Qingdao, Research Center for Intelligent and Wearable Technology, College of Textiles and Clothing, Qingdao University, Qingdao, China; ^2^ School of Environmental and Municipal Engineering, Qingdao University of Technology, Qingdao, China; ^3^ School of Biomedical Engineering, Health Science Center, Shenzhen University, Shenzhen, China

**Keywords:** flexible biosensor, point-of-care testing, polymer, paper, textile

## Abstract

With the outbreak and pandemic of COVID-19, point-of-care testing (POCT) systems have been attracted much attention due to their significant advantages of small batches of samples, user-friendliness, easy-to-use and simple detection. Among them, flexible biosensors show practical significance as their outstanding properties in terms of flexibility, portability, and high efficiency, which provide great convenience for users. To construct highly functional flexible biosensors, abundant kinds of polymers substrates have been modified with sufficient properties to address certain needs. Paper-based biosensors gain considerable attention as well, owing to their foldability, lightweight and adaptability. The other important flexible biosensor employs textiles as substrate materials, which has a promising prospect in the area of intelligent wearable devices. In this feature article, we performed a comprehensive review about the applications of flexible biosensors based on the classification of substrate materials (polymers, paper and textiles), and illustrated the strategies to design effective and artificial sensing platforms, including colorimetry, fluorescence, and electrochemistry. It is demonstrated that flexible biosensors play a prominent role in medical diagnosis, prognosis, and healthcare.

## Introduction

COVID-19 is a human infectious disease caused by novel coronavirus, which has posed a serious threat to the public health of more than 200 countries and regions around the world. Therefore, it is important to seek a rapid and accurate method to contain COVID-19 (Udugama et al., 2020). In general, traditional lab-based testing method requires not only bulky analyzers but also professionally operators. Furthermore, patients need to wait several days to get the test results due to the time-consuming preparation of reagents and samples. Point-of-care testing (POCT) allows medics to detect diseases at the patient site, faster than traditional lab-based testing, and plays an important part in the difficult times of the COVID-19 pandemic (Choi, 2020; Nelson et al., 2020; Sachdeva et al., 2020; Zhao et al., 2021a). Among the various devices of POCT, portable sensors with flexible substrates are the most attractive one (Economou et al., 2018).

Portable flexible biosensors represent an increasingly important and rapidly growing area of research (Guo et al., 2019; Manjakkal et al., 2020; Patil et al., 2020). There are some significant advantages of portable flexible biosensors: 1) more user-friendly, less affected by external conditions; 2) the test results can be obtained in time without waiting for a long time; 3) the production cost is low and it is beneficial to the resource-limited areas; 4) more suitable for wear, can be fitted to the skin to achieve more real-time sampling. At present, the vast majority of substrate materials come from polymers, perfect fabrication technology and abundant kinds of polymers provide strong support for the development of portable flexible sensors (Spychalska et al., 2020). Paper-based biosensors have received considerable interest in recent years owing to their advantages such as foldability, portability and accessibility (Lee et al., 2018a). Moreover, the surface of paper-based biosensors can be easily and quickly modified with biomolecules and nanomaterials, which is of great help to improve the sensing performance. The other important flexible biosensors use textiles as substrate materials, which have a promising prospect in the area of wearable sensors due to the knittable nature of the fabric (Farajikhah et al., 2019).

**FIGURE 1:**
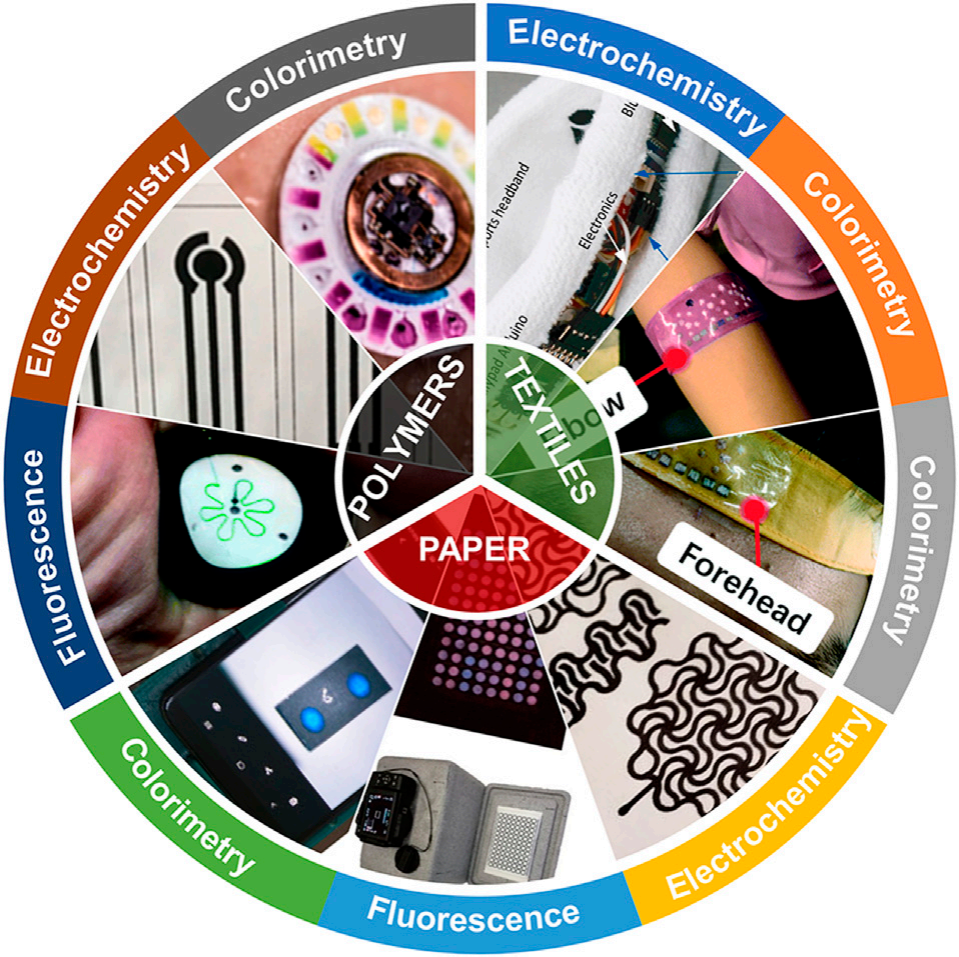
Schematic diagram of flexible biosensors based on colorimetry, fluorescence, and electrochemistry for point-of-care testing.

Here, as shown in Figure 1, we performed a comprehensive review of the applications of flexible biosensors in POCT based on the classification of substrate materials (polymers, paper and textiles). In addition, we also introduce the mainstream sensing strategies of flexible biosensors in detail, including colorimetric, fluorescent, electrochemical technology, etc. Finally, we summarized current problems and discussed the future development of the portable flexible biosensor. This review focuses on the important applications of flexible biosensors in POCT over the past 3 years and in order to help readers understand the future trends in this field.

## Polymer-Based Flexible Biosensors for POCT

Polymer substrates have been used in the printed electronics for a long time, such as polyethylene naphthalene (PEN), polyethylene terephthalate (PET), polyimide (PI), polytetrafluoroethylene (PTFE), polymethyl methacrylate (PMMA), polydimethylsiloxane (PDMS), polyaniline (PANI), polycarbonate and so on. Because of their inherent plasticity, wide availability, low cost, good insulative properties, and excellent elasticity, a variety of biosensors made of these carrier materials have been introduced one after another (Andrysiewicz et al., 2020; Lee et al., 2020; Wang et al., 2021).

### Colorimetric Test

Colorimetry is a common biochemical detection method, which is mainly used to evaluate the change of absorbance or reflectance of analytical reagents (Priye et al., 2018; Wang et al., 2019a). The basic requirement of colorimetric analysis is that the color reaction should have high sensitivity and selectivity, the colored compound generated by the reaction needs to maintain a relatively stable state. Therefore, providing a suitable reaction site and controlling the appropriate reaction conditions are the key to colorimetric analysis. These problems can be solved by emerging microfluidic technologies. Due to the difference of fluid performance between micro-scale and macro-scale, using micron pipes to control fluid can achieve a series of processing and treatment which are difficult to be completed by conventional methods. In recent years, microfluidic based POCT devices are widely used in the fields of chemical (Guo et al., 2018a), molecular biology (Yang et al., 2020) and biochemical analysis (Choi et al., 2018; Xu et al., 2018), while polymer substrates provide a broad stage for research and development of microfluidic technology.

**FIGURE 2:**
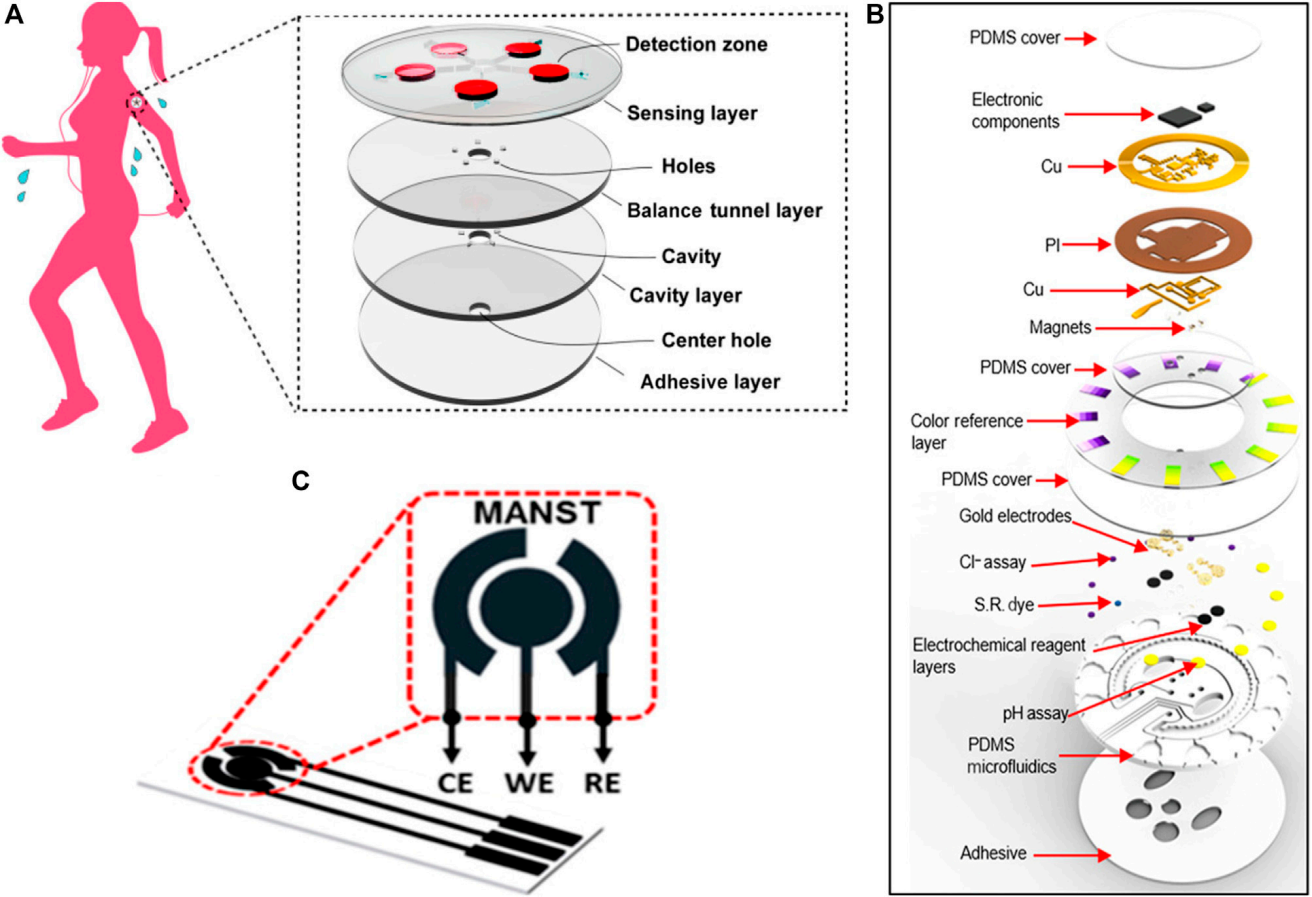
Biosensors based on polymer substrates. **(A)** The microfluidic substrate manufactured by PDMS consists of four layers that facilitate the flow of sweat from the skin surface to the top layer containing five assay chambers with pre-embedded colorimetric reagents (Xiao et al., 2019). **(B)** Exploded view of the complete PDMS-based hybrid battery-free platform (Bandodkar et al., 2019). **(C)** Electrodes printed on PET flexible substrates, contains working, counter and reference electrodes (Shaikh et al., 2019).

Sweat is an important metabolite, the physiological information contained therein can be easily monitored in a continuous and non-invasive way (Bariya et al., 2018). Xiao et al. (2019) reported on a wearable colorimetric sensor based on microfluidic chip made of PDMS that be used to detect glucose in sweat. The chip consisted of five microfluidic channels branching out from the center, and each of them can lead sweat from the epidermis to the detection microchambers (Figure 2A). Pre-embedded reagent in the microchamber will react with glucose in sweat and cause color change. Accordingly, this sensor can perform five parallel tests at the same time, greatly improving the detection efficiency. Due to the low modulus and high elasticity of the PDMS materials, the chip is flexible and can be stretched, twisted, and compressed without damage. Also based on PDMS materials, Bandodkar et al. designed a wearable sensing platform that combines advantages of electrochemical and colorimetric analysis (Figure 2B). The resulting sensor can monitor sweat rate, pH, lactate, glucose, and chloride at the same time (Bandodkar et al., 2019). Compared with the conventional sensors that only detect one single sample, this result provides a more comprehensive overview of physiological state.

Tear fluid is a complex fluid composed of lipids, electrolytes, proteins, peptides, glucose, and amino acids, sharing many similarities in composition with blood (Liu et al., 2019a). For example, the correlation between tear glucose and capillary blood glucose allows to determine the diabetic status of a subject using tear glucose values (Lin et al., 2018). POCT for tears has attracted great interest in recent years, but there are some difficulties in collection. Therefore, designing a device that can collect and analyze tear fluid in a timely manner is of great significance. Smart contact lenses have gained widespread interest for their ability to directly monitor physiological information in tears, and the primary concern in producing them is a suitable selection of flexible substrates. Acrylates can be self-polymerized or copolymerized with other monomers and are often used to make adhesives, rubber and plastics. Rosalia et al. have developed a sensor comes in the form of microfluidic contact lenses made of acrylate materials that can detect pH, glucose, protein and nitrite ions in tears (Moreddu et al., 2020a). In order to obtain accurate results, colorimetric readouts were performed using a smartphone-MATLAB algorithm based on the nearest neighbor model. Tested with artificial tears showed that the sensing device was able to respond within 15 s. An extension of this work by the same group has resulted in a new sensing platform by integrating paper microfluidics within laser-inscribed commercial contact lenses (Moreddu et al., 2020b), the device realized multiplex detection of clinically relevant analytes including proteins, glucose, nitrites, hydrogen ions and L-ascorbic acid.

### Fluorescence Test

Fluorescence analysis has a wide range of applications in biomedical research because of its high sensitivity, simple operation and strong specificity (Hou et al., 2019; Sun et al., 2021). Sekine et al. (2018) proposed a wearable sweat-analysis system for *in situ* quantitative analysis of sweat chemistry. The system includes a thin, soft microfluidic device and a smartphone-based optical module. The microfluidic device patterned on the PDMS consists of microchannel networks and microcontainers pre-filled with fluorescent probes. These probes react selectively with target analytes (chloride, sodium and zinc) in the sweat. Fluorescence intensity is detected by a smartphone, ultimately enabling quantitative and rapid analysis. According to experimental results in human subjects, the accuracy is equivalent to traditional laboratory technologies.

Hemophilia and albinism are both serious threats to human health. With the development of molecular biology, it is gradually recognized that these diseases are genetically related, so the application of DNA sensors in genetic testing is extremely valuable. DNA sensors can convert the information of target DNA into detectable electrical signals, which were developed on the basis of the interpenetration of various disciplines such as biology, chemistry, electronics technology and medicine (Srisomwat et al., 2021). Immobilization of the DNA probe is an important step in making a DNA sensor and determines its performance and quality (Huang et al., 2016; Huang et al., 2019). Daniso’s group modified the Polyethylene (PE) film surface using corona treatment, followed by the attachment of three different organo-silanes (Daniso et al., 2021). The results show that better adhesion of the DNA probe on the functionalized PE substrate significantly improved the biosensor’s limit of detection (LOD) to a value lower than 0.1 ng μl^−1^. Movilli et al. reported an effective method for selective modification of PDMS substrates utilizing modified poly-_L_-lysine (PLL) to enhance biomolecular adhesion (Movilli et al., 2019). By utilizing the electrostatic interactions between the positively charged modified PLL and the negatively charged polymer surface, a self-assembled layer is formed, bestowing the possibility of bio-orthogonal anchoring of various biomolecules. Three different peptide nucleic acid (PNA) probes printed as microarrays were able to selectively detect DNA from a mixture of DNA analytes, demonstrating the potential of the modified PLL approach for multiplexed detection of DNA sequences.

### Electrochemical Test

Electrochemical sensors achieve sensing by converting the chemical signal of the measured object into an electrical signal. Owing to their inherent miniaturization, low-power consumption, rapidity and low cost, POCT device with the electrochemical sensor meet the requirements of wearable sensing system (Christodouleas et al., 2018; Kanchi et al., 2018; Song et al., 2020). The flexible electrochemical biosensors can be directly attached to the skin surface and can maintain stable performance while undergoing mechanical deformation, which has been favored by researchers in recent years (Lee et al., 2018b; Kim et al., 2019; Seshadri et al., 2019). Preparation of electrodes is the foremost priority in the fabrication of electrochemical testing devices. A glucose sensor was fabricated by drawing gold electrode patterns on the surface of PET substrate via a chemical plating method. The sensitivity of the presented sensor was 22.05 μA mM^−1^ cm^−2^ in linear range of 0.02–1.11 mM and the LOD was 2.7 μM (Wang et al., 2019b). It exhibits good selectivity under the interference of lactic acid, uric acid, dopamine and ascorbic acid. Besides, it also shows excellent reproducibility and long-term stability for more than 1 month. Nanocomposites of graphene nanosheets with diblock copolymers and gold electrodes are proposed for the detection of prostate specific antigen (PSA) in saliva, and this sensing device demonstrated a wide range of PSA detection from 0.1 pgmL^−1^ to 100 ngmL^−1^ with LOD of 40 fgmL^−1^ (Khan et al., 2018). A screen-printed carbon electrode (SPCE) based on PET was reported for the assay of serum albumin (Figure 2C) (Shaikh et al., 2019). PANI/gold nanocrystals (AuNCs) nanocomposite films were modified on the surface of carbon electrodes, and the nanostructure of the PANI matrix provided a scaffold for the uniform distribution of AuNCs, resulting in an improved electrochemical response.

Electronic skin is a network of electronic sensors with mechanical deformation capabilities that are able to sense a variety of external stimuli like chemical, mechanical and thermal stimuli (Heikenfeld et al., 2018; Xu et al., 2019; Yang and Gao, 2019). Lee’s group presented an electronic skin capable of converting mechanical stimuli into visual readouts (Lee et al., 2021). The material layer of the electronic skin is made of a physically cross-linked polymer matrix with a mixture of ionic transition metal complexes (ITMC) and ionic liquids (IL) encapsulated in it. There is a visco-poroelastic response of the material platform to mechanical stress that causes a change in the distribution of ionic luminophore in the film, which enables the visualization of the stresses applied to the electronic skin.

Molecularly imprinted polymer (MIP) is a special type of polymers with specific recognition and selective adsorption that is synthesized using molecular imprinting technology (MIT). MIT was developed on the basis of bio-nanotechnology by simulating enzyme-substrate and receptor-antibody interactions, and chemists commonly use this technology for molecular recognition studies (Tricoli and Neri, 2018; Ahmad et al., 2019; Lowdon et al., 2020). An ultrasensitive MIP based on carbon screen printed electrodes (C-SPE) was presented for the first time to detect interleukin-6 (IL-6), the Alzheimer’s disease (AD) biomarker (Gonçalves et al., 2021). The polymer was electro-synthesized from pyrrole and carboxyl pyrrole under near physiological conditions. IL-6 was trapped in the polymer matrix and subsequently removed by acid cleavage to generate the imprinted sites. This biosensing material showed promising results for POCT of neuroinflammatory biomarkers, which is valuable for the development of AD prevention.

## Paper-Based Flexible Biosensors for POCT

Paper, composed primarily of cellulose, is ubiquitous in our society and widely used for writing and drawing. However, they hold an equally promising research value in the emerging field of flexible sensors (Wang et al., 2018; Aydindogan et al., 2020). Compared with traditional substrate materials like silicon, glass and rigid polymers, paper has many significant advantages for the fabrication of bioanalytical devices, such as excellent flexibility, low price, and light weight. In addition, the most attractive aspect of cellulose is from its porosity, which provides the paper with good hydrophilicity and allows it to be used as a natural microfluidic system.

### Colorimetric Test

Deficiency of glucose-6-phosphate dehydrogenase (G6PD) in red blood cells is one of the causes of neonatal jaundice. White et al. proposed a paper-based biosensor for visual detection of G6PD, which is quite simple to fabricate and does not require any complex instrumentation (White et al., 2020). The paper biosensor performs G6PD detection through the chromogenic effect generated by the catalysis of dehydrogenase. In addition, the sensor has an ingenious design with all critical reagents contained on the paper or in the diluent buffer, thus reducing the operation steps by users. Similarly, for the prevention of neonatal jaundice, a tape-paper-based sensing device designed and manufactured by Tan et al., which is able to separate plasma from whole blood samples and measure total bilirubin using a colorimetric diazotization method (Tan et al., 2020). The tape-paper sensing method overcomes non-homogeneous color distribution caused by the deposition of analyte, indicator and reaction product at the edge of the reaction zone, and improves the accuracy of colorimetric evaluation of paper-based analytical equipment.

The development of nanomaterials (carbon nanotubes, metal nanoparticles, graphene and quantum dots) for biosensors has been broadly reported in recent years (Lim et al., 2015; Nguyen and Kim, 2020; Wang et al., 2020). Nanomaterials can be used as signal markers or carriers of signal markers for sensitive and specific detection of biomolecules, in addition, a number of nanomaterials with excellent optical properties have been widely applied in colorimetric analysis (Aydindogan et al., 2018). The conjunction of nanomaterials and portable biosensors have acquired numerous applications (Hui et al., 2018; Ji et al., 2018; Faham et al., 2019), an interesting example is the gold nanoparticle-based paper sensor capable of detecting 11 benzimidazoles simultaneously, proposed by Guo’s group (Guo et al., 2018b). The immunochromatographic strip is assembled and labeled with gold nanoparticles, and detection results are available in just 15 min. Polydiacetylene (PDA) is a commonly used conjugated polymer with unique chromatographic properties for sensitive optical transduction. PDA nanovesicles with particle size below 100 nm facilitate higher sensitivity owing to their larger surface area for reaction. In addition, a PDA vesicle-based paper strip was recently described, and smartphone was used for image analysis, enabling rapid and quantitative analysis of influenza A (pH1N1) virus at low concentrations (Son et al., 2019). It was confirmed that the LOD of PDA paper strip is from 5 × 10^3^ to 10^4^ TCID50 (50% tissue culture infective dose). Metal-organic framework (MOF) materials are coordination polymers with a three-dimensional pore structure, normally connected by metal ions and supported from organic ligands, which have been developed rapidly in the last decade and shown wide applications in catalysis, energy storage and sensing. ZIF-8 is a nano-scale MOF material, Kou et al. demonstrated a portable, user-friendly smartphone-assisted biomimetic MOF nanoreactor colorimetric paper (SBMCP) for POCT of endogenous biomolecules (Kou et al., 2020). Inspired by intracellular cascade signal transduction, the enzyme component trapped in the ZIF-8 exoskeleton allowed for selective and sensitive recognition of target analytes via micropores, followed by conversion of the recognition information into a visual color signal through the cascade reaction. Additionally, the ZIF-8 frameworks can significantly improve the stability of enzyme.

### Fluorescence Test

**FIGURE 3:**
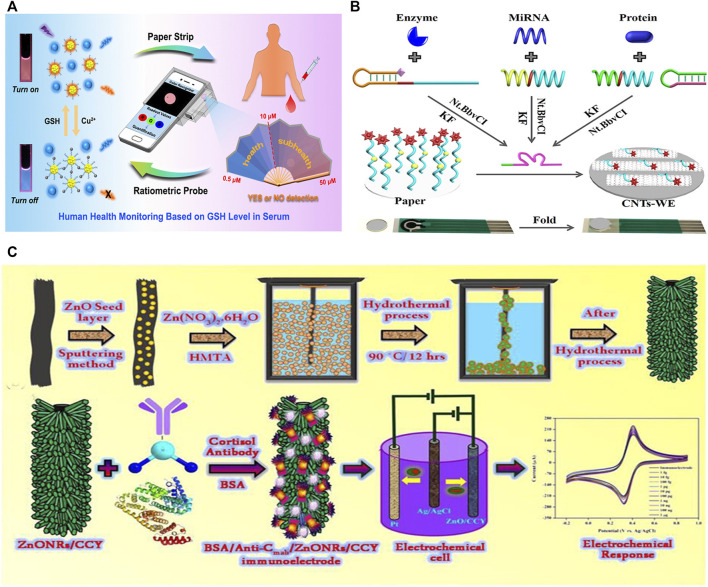
Biosensors based on paper and textile substrates. **(A)** Schematic diagram of a fluorescent paper strip integrated with a portable smartphone platform for monitoring glutathione in human serum (Chu et al., 2020). **(B)** Specially designed universal paper-based electrochemical sensor (Liu et al., 2019b). **(C)** Schematic diagram of a zinc oxide nanorod electrochemical sensing electrode integrated with flexible carbon fibers for sweat cortisol detection (Madhu et al., 2020).

Three-dimensional (3D)-printing technology is actually an additive manufacturing method based on light-curing and paper lamination technology that can turn blueprints in a computer into physical objects in an easy way. Nowadays, 3D-printing is commonly used to manufacture prototypes and gradually gaining prominence in industrial design, aerospace, and medical fields (Scordo et al., 2018). Combined with 3D-printing, Chu et al. (2020) introduced a portable smartphone-based quantitative fluorescence sensing paper strip, capable of accomplishing rapid, real-time, reliable and on-site determination of glutathione (GSH) in human serum, which shows great significance for early disease diagnosis. The sensitive fluorescent nanoprobe was constructed through the mixture of blue carbon dots (CDs) and orange gold nanoclusters (AuNCs). The blue fluorescence remained stable in the presence of copper ions, while the AuNCs with orange fluorescence were quenched (Figure 3A). Upon the addition of GSH, the quenched orange fluorescence could be quickly restored, displaying a visible color change from blue to orange, due to the stronger affinity between copper ions and GSH. It was verified that the LOD was 1.84 μM, which is far below the lowest level of GSH in serum.

The fluorescent intensity change that occurs when the spectra of donor and acceptor fluorescent molecules overlap is called fluorescence resonance energy transfer (FRET). Bioluminescence resonance energy transfer (BRET) is one of the FRET, which donor luminophore is a bioluminescent molecule and its electrons are excited by a bioluminescent chemical reaction (Tomimuro et al., 2020). This section will present several interesting applications of BRET in portable paper-based sensors. Yu et al. (2018) created a semi-synthetic luminescent sensor protein that can accurately quantify metabolites in blood by paper-based analysis. The device consists of test paper containing the lyophilized sensor, a cardboard box that provides dark environment, and a digital camera used to capture the fluorescent signal. The metabolite is oxidized by the nicotinamide adenine dinucleotide phosphate (NADPH) on the test paper and the sensor changes color, realizing metabolite quantification. Phenylalanine levels in the blood of patients can be obtained in minutes with this sensor, and the difference is less than 15% compared to conventional assays. Tenda et al.’s work reported on microfluidic paper-based assay devices (μPADs) that rely on BRET-based antibody-sensing proteins for analyte recognition and colorimetric signal generation, which achieved simultaneous detection of three antibodies in whole blood (Tenda et al., 2018). The enclosed space created by vertically assembled and fully laminated paper layers can limit sample volume absorbed by the paper, allowing for sample volume-independent measures. In view of its simplicity, this device is well suited for POCT in resource-limited areas.

### Electrochemical Test 

Among the paper-based sensor signal readout schemes, electrochemical strategies are more attractive compared to optical methods because of better quantitative and interference immunity. However, high background currents have been a persistent problem faced by electrochemical methods, which results in a loss of sensitivity. To solve this problem, Liu et al. tried to immobilize signal molecules on paper separated from the working electrode, which cannot diffuse or adsorb to the electrode surface non-specifically without target induction (Figure 3B) (Liu et al., 2019b). With that in mind, a paper-based electrochemical sensor was fabricated, which can be used for highly sensitive analysis of various different biomarkers. An electrode was designed for the detection of 3-nitrotyrosine (3-NT) in urine (Martins et al., 2020). The MIP for 3-NT was modified on a paper platform and conducted with carbon ink. After condition optimization, the linear detection range of the label-free sensor for 3-NT is 500 nM–1 mM, and the detection limit is evaluated to be 22.3 nM. Mohammadifar et al. (2019) demonstrated a multi-threshold glucose detection test strip that can detect five different glucose concentration levels. The five electrodes integrated in the paper strip were modified with glucose oxidase, and the indicator circuitry will generate a reliable visual signal when a predetermined glucose concentration is reached.

Pencil and paper are wonderful partners in writing and drawing. However, their applications extend beyond that, as the pencil-paper-based on-skin electronic devices designed by Xu et al. (2020) explored applications in emerging skin interface health monitoring and interventions. In this work, a commercial 9B pencil and commonly available office copy paper are the main elements that make up this biosensor. Pencil-drawn graphite patterns are used as conductive traces and sensing electrodes, and paper acts as flexible substrates. Various on-skin electronic sensors were designed by drawing different pencil patterns, including biophysical sensors and sweat biochemical (pH, uric acid, glucose) sensors. Notably, the pencil-paper-based uric acid (UA) sensor has a high sensitivity of 21.9 nAμM^−1^ in the 0–600 μM concentration range, exhibiting comparable performance to conventional sensors based on carbon materials.

Foldability is a unique feature of paper, some paper-based sensors take advantage of foldability to achieve highly integrated, which makes it easy to reduce the sensor size. Li et al. (2021) designed a sensing paper for wearable electrochemical sweat analysis utilizing 2D Ti_3_C_2_T_x_ as the active materials and foldable paper substrates as sweat analysis patches. Four functional layers are printed on different areas of the paper substrate to facilitate the decoration of enzymes and electrochemical materials, and then fold into 3D structure. The unique 3D structure boosts sweat absorption, improves the accuracy of detecting sweat components, and reduces discomfort caused by sweat accumulation at the human-machine interface. Such a wonderful structure was utilized to prepare a dual-channel sensor, which can detect glucose and lactate simultaneously with a sensitivity of 2.4 nA μM^−1^ and 0.49 μA mM^−1^, respectively. A portable, robust and user-friendly cobalt-MOF modified carbon cloth/paper (Co-MOF/CC/Paper) electrochemical sensor was fabricated for non-enzymatic quantitative glucose detection (Wei et al., 2021). Co-MOF was crystallized on CC to successfully manufacture a highly integrated electrochemical analytical chip that effectively increased the specific surface area compared to conventional planar electrodes. The chip assembled with patterned paper electrodes and finally folded into a fabulous Co-MOF/CC/paper hybrid button-sensor. The introduced button structure allows easy separation of the working electrode from the sample region and facilitates sample replacement.

## Textile-Based Flexible Biosensors for POCT

Textiles are a fascinating material. Countless individual fibres are interlaced in different manufacturing steps, culminating in a complex structure with quite decent properties. No other material combines drapability, lightness, robustness and decorative properties. However, textiles have been discovered to possess more than just these properties, and recently high-performance textiles have demonstrated amazing examples in military and aerospace applications, such as carbon fibre, aramid and other materials applied to soldiers’ protective clothing, which not only improve defensive performance, but also increase warfare efficiency. (Kirstein, 2013). If textiles are capable of such high-tech capabilities, it is logical to consider them for portable wearable sensing applications (Hu et al., 2020).

### Colorimetric Test

Urine is a liquid excretion that includes haemoglobin, enzymes, and electrolytes in its composition. In the medical field, urine is a frequently used sample and urinalysis could help physicians diagnose many diseases. To further exploit the advantages of non-invasiveness and accessibility, various POCT devices for urine testing have emerged in recent years (Alves and Reis, 2019; Michael et al., 2020). Integrating a tape-based sensor with a diaper, He et al. (2020) introduce a wearable biosensor for fast multiplex urinalysis. The micro-sensing array was arranged on a flexible waterproof tape through physical punching and textile filling processes, and then adhered to the diaper. One of the major advantages of this tape-based sensor is that urine can be sampled and detected directly through the diaper, without any additional operations in the whole process. This device enables the detection of urinary markers such as glucose, nitrite, protein and phenylpyruvate, which are important for the early warning of some diseases, representing an ideal POCT solution for infants and urinary incontinence patients. A similar example of wearable biosensors could also be seen in sweat detection, Zhao et al. (2021b) prepared a wearable microfluidic thread/fabric-based analytical device (μTFAD) for perspiration sensing. Hydrophilic patterns and a hydrophobic surface were delineated by embroidering cotton threads on a hydrophobic treated cotton cloth. In this case, the hydrophilic parts served as channels and detection zones, which were treated with colorimetric indicators. Smartphone assisted readings showed LOD was 10 μM for glucose concentration, 10 mM for chloride concentration and 4.0–9.0 for pH.

Nanozymes provide not only the high catalytic activity of native enzymes, but also the stability and cost-effectiveness of model enzymes, which are widely accepted in medical, chemical, food and agricultural fields. Reliable glucose testing in the urine is essential for the prevention of diabetes, Karim’s team, for the first time, demonstrated silver (Ag) nanoparticles embedded in cotton fabric as free-standing nanozymes for the rapid assay of glucose in urine (Karim et al., 2018). Using cotton as a substrate provides a large number of catalytic active sites for the catalytic reaction, and the excellent absorption characteristic of cotton fibres also facilitates the rapid absorption of glucose. Following the similar principle, Karim et al. also attempted to embed catalytically active copper (Cu) nanoparticles into cotton fabrics to develop a glucose monitoring system (Naveen Prasad et al., 2021). The sensor could operate in the 0.5–15 mM glucose concentration range and provides minimal sample matrix effects, allowing almost direct detection of glucose from urine without complex sample processing.

### Electrochemical Test

Mechanical flexibility is critical for a wearable biosensing platform because it is related to the stability and accuracy of the biosensor in real use. In this sense, the importance of designing electrodes with stable tensile performance under mechanical deformation can hardly be overemphasized. An example of a wearable electrochemical biosensor on the basis of flexible zinc-oxide nanowires (ZnO NWs) electrodes is described, which could be applied to the detection of lactate and sodium in sweat during exercise (Zhao et al., 2021c). Thread-based carbon electrodes were fabricated from cotton thread, PDMS, carbon ink, silver and silver chloride (Ag/AgCl) ink, while ZnO-NWs were grown *in situ* on the electrodes by a hydrothermal process. Cotton threads were wrapped around the lactic acid sensing electrode and sodium sensing electrode, in order to assist in sweat absorbing, thus providing reaction reservoirs and transfer routes for lactic acid and sodium ions.

Due to the special structure of graphite microcrystals, carbon fibers possess high strength and modulus along the fiber axis direction. In addition, carbon fibers possess favorable conductivity and have a promising future in the field of textile-based sensors. Madhu’s group proposed a flexible yarn-based electrochemical immunosensing method for the analysis of sweat cortisol (Madhu et al., 2020). As shown in Figure 3C, ZnO nanorods (ZnONRs) were coated onto flexible carbon yarns using a hydrothermal treatment to facilitate the immobilization of specific anti-cortisol antibodies. The network structure of carbon fibers offers a larger surface area for electrocatalytic reactions, enabling highly sensitive and selective sensing, and making them the prime substrates in biosensor platforms. The sensor showed a range of linear detection from 1 fg ml^−1^ to 1 μg ml^−1^, and the LOD was between 0.45 and 0.098 fg ml^−1^ obtained by Cyclic Voltammetry (CV) and Differential pulse voltammetry (DPV) techniques.

## Conclusion and Outlook

In the past decades, POCT has brought constant surprises. In this review, we systematically review the application of various biosensors in POCT in recent years, categorized by different substrates, in combination with the corresponding sensing methods. These sensing devices overcome the limitations of traditional laboratory tests effectively and present significant potential, especially in resource-limited areas. Polymers as the most commonly used substrate materials in flexible sensors currently, have driven the advancement of microfluidic technology in POCT. Microfluidic-based POCT aims to obtain results faster in order to improve the efficiency of disease detection and the accuracy of diagnosis. Furthermore, using small sample volumes greatly saves the expense of reagent consumption. However, contamination may occur from the production of polymers, which is the most criticized problem. Paper-based sensors have recently become the focus in the construction of portable flexible biosensing platforms. The foldable behavior of paper allows for many marvelous ideas in sensor fabrication, for example, light weight cellulose paper with excellent porosity can be designed as a template for highly integrated microfluidic sensors just by simple folding. The simplicity of fabrication steps is another attractive advantage, which also leads to the low cost of paper-based sensors. But as an emerging technology, paper-based sensors suffer from lack of stability and poor sensitivity. Although integrating sensors with textiles is cumbersome and subject to the problems of short service life and poor robustness, it is undeniable that manufacturing sensors on textiles can improve the user-friendliness of wearable sensors, because they are constantly and intimately in contact with the human body in daily life. The wicking property of fabrics is important which provides a pathway for capturing and transporting target biomolecules to the sensor surface, and it is believed that textile-based sensors will play a key role in detecting biomarkers within body fluids.

Although there are still gaps in the accuracy and stability of POCT compared with traditional laboratory-based tests, the COVID-19 pandemic underscores the importance of more rapid testing platforms. Overall, the opportunities and challenges facing POCT are enormous, making early commercialization still pending the concerted efforts of researchers.
