# Corrigendum: Flexible biosensors based on colorimetry, fluorescence, and electrochemistry for point-of-care testing

**DOI:** 10.3389/fbioe.2022.1087738

**Published:** 2022-11-15

**Authors:** Tingyi Yan, Guangyao Zhang, Huining Chai, Lijun Qu, Xueji Zhang

**Affiliations:** ^1^ State Key Laboratory of Bio-Fibers and Eco-Textiles, Intelligent Wearable Engineering Research Center of Qingdao, Research Center for Intelligent and Wearable Technology, College of Textiles and Clothing, Qingdao University, Qingdao, China; ^2^ School of Environmental and Municipal Engineering, Qingdao University of Technology, Qingdao, China; ^3^ School of Biomedical Engineering, Health Science Center, Shenzhen University, Shenzhen, China

**Keywords:** flexible biosensor, point-of-care testing, polymer, paper, textile

In the published article, there was an error in Affiliation 1. Instead of “State Key Laboratory of Bio-Fibers and Eco-Textiles, Intelligent Wearable Engineering Research Center of Qingdao, Research Center for Intelligent and Wearable Technology, College of Textiles and Clothing, Qingdao, China,” it should be “State Key Laboratory of Bio-Fibers and Eco-Textiles, Intelligent Wearable Engineering Research Center of Qingdao, Research Center for Intelligent and Wearable Technology, College of Textiles and Clothing, Qingdao University, Qingdao, China.”

The authors apologize for this error and state that this does not change the scientific conclusions of the article in any way. The original article has been updated.

